# Place for elective cholecystectomy for patients with severe thalassaemia: a retrospective case control study

**DOI:** 10.1186/s13104-019-4285-1

**Published:** 2019-04-29

**Authors:** Anuja Premawardhena, Roshanthi Fernando, Sumudu Kumarage, Nilanga Nishad, Dilith Goonatilleke, Ishari Silva, Sachith Mettananda

**Affiliations:** 1 Department of Medicine, Faculty of Medicine, University of Kelaniya, PO Box 6, Talagolla Rd., Ragama, Sri Lanka; 2 North Colombo (Teaching) Hospital, Ragama, Sri Lanka; 3 Department of Surgery, Faculty of Medicine, University of Kelaniya, Ragama, Sri Lanka; 4 Department of Paediatrics, Faculty of Medicine, University of Kelaniya, Ragama, Sri Lanka

**Keywords:** Thalassaemia, Elective cholecystectomy, Timing of cholecystectomy

## Abstract

**Objective:**

At present, cholecystectomy is carried out for thalassaemia patients with gall stone disease only if they develop symptoms of cholecystitis, except in the rare instance where an un-inflammed gall bladder is removed simultaneously with splenectomy. We carried out this retrospective analysis of case records to examine if patients with thalassaemia have a higher rate of peri operative complications compared to non-thalassaemics with gall stone disease, warranting a change of policy to justify elective cholecystectomy.

**Results:**

Case records of 540 patients with thalassaemia were retrospectively analysed of which 98 were found to have gallstones. Records of 62 patients without thalassaemia with gall stone disease too were used for comparison. 19 of patients with thalassaemia and 52 of non-thalassaemic who had gallstones had undergone cholecystectomy. In all but 5 patients with thalassaemia cholecystectomy was done following attacks of acute cholecystitis as was the case in the non-thalassaemic controls. A significantly higher proportion of early and late complications had occurred in thalassaemia patients compared to non-thalassaemic patients post operatively. Six deaths related to sepsis following acute cholecystitis in the peri operative period were reported among 19 thalassaemia patients whereas no deaths were reported among 55 non-thalassaemic patients who underwent cholecystectomy for gallstones.

## Introduction

Haemoglobinopathies including thalassemia are commonest monogenic disorders in the world [1]. It is estimated that approximately 7% of the world population are carriers for a haemoglobin disorder and 300,000 children with severe forms of haemoglobinopathies are born each year [1, 2].

Gallstone formation is a known complication of all types of chronic haemolytic anaemias including thalassemia. Co-inheritance of thalassaemia with inherited disorders of bilirubin metabolism, particularly Gilbert syndrome, increases the risk of gallstone formation [3]. Gilberts syndrome and haemoglobinopathies both reach high population prevalence in similar regions in the world including the South Asian subcontinent [4].

In the normal population, approximately 10–20% of people develop gallstones, mostly in their middle ages. The majority of them remain asymptomatic for many years and progression to symptomatic disease occurs only in approximately 10–20% [5]. Gallstones in non-thalassaemic individuals are left un-operated until they become symptomatic as it is felt that gallstones will not cause harm unless they are complicated with cholecystitis or obstruction. At present, a similar approach is applied to thalassaemic patients when gallstones are detected on routine scanning, and they are offered surgery only if they develop symptoms referable to the gall bladder or the duct system. However, this approach has raised concerns. Patients with thalassaemia develop gallstone disease at a younger age and are likely to have several cardiac, hepatic and endocrine co-morbidities compared to non-thalassaemic individuals. It is therefore possible that patients with thalassaemia are more likely to develop complications of gallstones than their non-thalassaemic counterparts. Therefore, management of gallstones in patients with thalassaemia requires a disease-specific and evidence-based approach.

We were unable to find any studies which address the issue of timing of cholecystectomy for thalassaemia. As a prologue to a prospective study on this issue, we set out to do a retrospective case analysis of thalassaemia patients who underwent cholecystectomy, specifically to look at the complications they may have developed during the peri operative period.

## Main text

### Methods

The aim of this study is to describe the frequency of gallstones among patients with thalassaemia and to compare the profiles of comorbidities and peri-operative complications following cholecystectomy among patients with gallstones with and without thalassaemia. A retrospective study was carried out at the Colombo North Teaching Hospital (CNTH), Ragama and Teaching Hospital (TH), Kurunegala using clinical records of patients with thalassemia and patients without thalassaemia during a 6-month period started from May 2010. Ethical and administrative permission for the study was granted from the Ethics Review Committee Faculty of Medicine University of Kelaniya as well as the two institutions concerned. CNTH is one of the eight University Hospitals and is the main tertiary referral centre for parts of the Western Province and TH Kurunegala is the only tertiary care hospital for the North-Western Province of Sri Lanka. These two hospitals manage the two largest thalassaemia centres of Sri Lanka which care for approximately 40% of all patients with thalassaemia in the country. Patient records of all patients with thalassaemia and other chronic anaemias attending for regular blood transfusions to the thalassaemia centres were studied consecutively. All accessible records of patients were considered for perusal. After excluding the records of patients of sickle cell disease, sickle cell β-thalassaemia and hereditary spherocytosis, 540 patient records were selected for analysis. The information was recorded in a pre-formed questionnaire developed and filled by the first author. Data on present and past medical problems, investigations and details of routine annual abdominal ultrasonography were extracted using a data collection form by the principal investigator to identify thalassaemia patients with gallstones (98 out of 540). In order to compare the peri-operative and postoperative morbidity and mortality of cholecystectomy among thalassaemia (n = 19), we perused patient records of non-thalassaemia patients who were diagnosed to have gallstones or referred to the hospital for gallstones on the same time period at CNTH and Teaching Hospital, Kurunegala (n = 62). Both the thalassaemia patients as well as the non-thalassaemia “controls” were operated by the same two surgical teams in the two hospitals. Both teams were led by a specialist general surgeon with similar qualifications and approximately similar experience. Further data related to symptomatology as well as cholecystectomy (if undergone, n = 19) and post-operative complications following cholecystectomy (if present) were recorded in the subset of patients who had gallstones (n = 98). Post-operative complications such as liver abscess and sepsis occurring within the first week (day 0–7) following surgery were defined as early complications whilst complications developing after 7 days were defined as late complications.

Socio-demographic details and past medical history of the gall stone patients with and without thalassaemia were compared initially and peri-operative, post-operative complications were compared in cholecystecomized patients in those two groups. Categorical variables were expressed as counts and percentages and compared using odds ratios and 95% confidence intervals. Continuous variables were expressed as mean with standard deviation and are compared using independent sample Student’s t-test. Ethical approval was obtained from the Ethics Review Committee of the Faculty of Medicine, University of Kelaniya.

### Results

A total of 540 patients with thalassaemia and chronic anaemias were recruited into the study (age range—3 months to 55 years). Out of them, 98 (18.1%) had gallstones. A majority of the patients [54 (55%)] with gallstones had Haemoglobin E (HbE) β-thalassaemia whilst 29 (30%) had β-thalassaemia major. Six (6%) patients had β-thalassaemia intermedia and the remainder (9%) had non thalassaemic conditions like G6PD deficiency.

#### Comparison of thalassaemia patients with gallstones and non-thalassaemic patients

Next, we compared the demographic and co-morbidity profiles of thalassaemia patients with gallstones and 62 non-thalassaemic patients with gallstones (Tables 1 and 2). The proportion of females in the non-thalassamic group were significantly higher compared to thalassaemia group. Similarly, patients with thalassaemia developed gallstones at a significantly younger age (mean 26.8 ± 10.9 years) compared to non-thalassaemic patients (mean 47.5 ± 19.7 years) (t = 8.54, p < 0.0001).

**Table 1:** Main demographic descriptors of the two groups with gall stones

	Thalassaemia patients with gall stones	Non thalassaemia patients with gall stones
Number	98	62
Gender (females)	45 (46%)	47 (76%)
Mean age (years)	26.8 ( ± SD 10.9)	47.5 ( ± SD 19.7)
Median age (IQR) (years)	24.2 (18.2–29.2)	44.7 (39.7–49.7)

**Table 2:** Demographic and co-morbidity profile of patients with gallstones with or without thalassaemia

Demographic/medical factor	Number (%) of thalassaemia patients with gallstones having associated factor (n = 98)	Number (%) of non-thalassaemia patients with gallstones having associated factor (n = 62)	Odds ratio	95% CI	p value
Female sex	45 (46%)	47 (76%)	0.27	0.13–0.54	< 0.001
Diabetes mellitus	10 (10%)	14 (22%)	0.38	0.16–0.94	< 0.05
Dyslipidaemia	0 (0%)	9 (14%)	0.03	0.001–0.50	< 0.05
Cirrhosis	8 (8%)	0 (0%)	11.7	0.66–207.1	0.09
Hypothyroidism	14 (14%)	0 (0%)	21.4	1.25–366.4	< 0.05

Patients with thalassaemia had significantly higher incidence of hypothyroidism and lower incidences of diabetes and dyslipidaemia compared to non-thalassaemia patients with gallstones. None of the patients had co-existing ischemic heart disease, iron-related cardiomyopathy or cardiac failure.

Nineteen (19%) out of the 98 patients with thalassaemia who had gallstones had undergone cholecystectomy. Fourteen of them had surgery because they developed symptoms related to gallstones. Remaining 5 patients who underwent cholecystectomy without symptoms had their gall bladders removed simultaneous during splenectomy in an attempt to prevent further surgery. Out of the 62 non-thalassaemic patients with gallstones, 55 (88%) underwent cholecystectomy. A majority (96.3%) of non-thalassaemic patients underwent laparoscopic cholecystectomy, whilst a majority (68%) of patients with thalassaemia underwent open cholecystectomy. In five patients this was due to the cholecystectomy been done concomitantly with splenectomy.

Next, we looked at the rate of post-operative complications following cholecystectomy in both groups (Table 3). A significantly higher proportion of early and late complications occurred in thalassaemia patients compared to non-thalassaemic patients. Among early complications, surgical site pain and wound infections were not significantly different in the two groups however, sepsis and liver abscess formation were common among patients with thalassaemia (Table 3). Recurrent abdominal pain was more common among the thalassaemics as a late complication.

**Table 3:** Post-operative complications following cholecystectomy

Demographic/medical factor	Number (%) of thalassaemia patients who developed the complication (n = 19)	Number (%) of non-thalassaemia patients who developed the complication (n = 55)	Odds ratio	95% CI	p value
Early	8 (42.1%)	10 (18.1%)	3.27	1.04–10.2	< 0.05
Sepsis	2 (10.5%)	1 (1.8%)	6.35	0.54–74.4	0.14
Liver abscess	1 (5.2%)	0 (0%)	9.0	0.35–230.6	0.18
Late (recurrent abdominal pain)	6 (31.5%)	7 (12.7%)	3.16	0.90–11.0	0.07

Six deaths were reported among 19 thalassaemia patients who underwent cholecystectomy due to gallstones. In the death records of five of them the deaths were directly attributed to acute cholecystitis while the death of one patient following cholecystectomy was attributed to sepsis and heart failure however, no post mortem data were available in any of these patients. There were no peri-operative deaths among non-thalassamic patients who underwent cholecystectomy for gallstones. Importantly no complications were noted in any of the five patients who underwent elective cholecystectomy at splenectomy.

### Discussion

Though gallstone formation is a well-known complication of chronic haemolytic disorders its, is not widely considered as a major health problem among patients with thalassaemia. Results of our study show that this concept needs to be revised. The prevalence of gallstones in our population was as high as 18%. This figure is lower than to the prevalence of gallstones among patients with sickle cell disease and congenital spherocytosis which report prevalence of gallstones of 30% and 21–63% respectively [6, 7].

Elective cholecystectomy is not offered at present to thalassaemia patients who are found to have asymptomatic gallstones on routine abdominal ultrasonography. Surgery is currently indicated only if they develop symptoms related to gallstones, acute cholecystitis in particular. However, our results show that a significant proportion (19/98; 19.3%) of thalassaemia patients with gallstones required cholecystectomy. A majority of them (14/19; 73.6%) required cholecystectomy following attacks of acute cholecystitis. Unlike with those without thalassaemia, acute cholecystitis in thalassaemic patients can lead to life threatening complications. In our cohort, five out of six deaths among patients with gallstones were due to acute cholecystitis. It is possible that these deaths could have been prevented if cholecystectomy was performed in patients with thalassaemia and gallstones electively before acute cholecystitis developed.

Similar observations were recently made in patients with sickle cell diseases. Several studies suggest the benefit of laparoscopic elective cholecystectomy for gallstones in asymptomatic children with sickle cell disease which have led to lower the threshold for laparoscopic cholecystectomy in patients with asymptomatic gallstone disease [8]. This recent shift of policy towards elective laparoscopic cholecystectomy was affected by the understanding of associated sickling crises and complications associated with cholecystitis in patients with sickle cell disease, together with the increased safety of laparoscopic procedures.

Another striking observation of this study is the higher incidence of post-operative complications following cholecystectomy among patients with thalassaemia compared to controls. There seems to be a significantly higher rate of severe sepsis associated complications in them. This could partly be explained by higher proportion of thalassemia patients undergoing open cholecystectomy as opposed to laparoscopic procedures however, could also be due to other co-morbidities among them. Nonetheless we believe that this should also support our opinion that the cholecystectomies should be performed electively among patients with thalassaemia who have gallstones.

### Conclusions

In conclusion, this study identified higher incidence of peri-operative complications following cholecystectomy among patients with thalassaemia which is most likely due to the fact that surgeries were performed following attacks of cholecystitis. We feel that there is a strong case to consider elective laparoscopic cholecystectomy in patients with thalassaemia to prevent the development of severe complications associated with cholecystectomy when symptomatic. We hope that the results of this study would make the haemoglobinopathy care physicians more aware about this largely under researched area in patients with thalassaemia. Further studies should be ideally prospectively designed utilising the same set of surgeons using similar surgical techniques so that outcome comparison can be done without reservation.

## Limitations

This is a retrospective study performed by perusing patient records. A major obstacle we faced was lack of post mortem data which made it extremely difficult to accurately identify the cause of death in the six individuals who died during the peri-operative period. However, available death certificates indicated cholecystitis as the cause of death in all of them.

The usage of two surgical procedures, namely laparoscopic cholecystectomy as well as open cholecystectomy confounds the results. The majority of the thalassaemics undergoing open surgery as opposed to the non thalassaemics undergoing laparoscopic procedures prevents an accurate comparison of perioperative complications. The reasons for the choice of surgery was not specified in the patient notes and could not be used in the analysis.

Despite limitations, to the best of our knowledge, this is the first study which evaluates and compares symptomatology, comorbidities and complications of thalassaemia patients with gallstone with non-thalassaemia controls.

## Abbreviations

CNTH: Colombo North Teaching Hospital
TH: Teaching Hospital
